# Effects of robotic walking on bone and muscle health in youth with neuromotor impairments: a five-participant case series

**DOI:** 10.3389/fendo.2026.1916359

**Published:** 2026-09-23

**Authors:** Erin Hodgson, Jessica L. Youngblood, Christa M. Diot, Benjamin M. Norman, Leigh Gabel, Elizabeth G. Condliffe

**Affiliations:** 1 Biomedical Engineering Graduate Program, University of Calgary, Calgary, AB, Canada; 2 Faculty of Kinesiology, University of Calgary, Calgary, AB, Canada; 3 McCaig Institute for Bone and Joint Health, University of Calgary, Calgary, AB, Canada; 4 Alberta Children’s Hospital Research Institute, University of Calgary, Calgary, AB, Canada; 5 Departments of Clinical Neurosciences and Pediatrics, University of Calgary, Calgary, AB, Canada; 6 Hotchkiss Brain Institute, University of Calgary, Calgary, AB, Canada

**Keywords:** adolescents, bone, cerebral palsy, children, muscle, neuromotor impairments, pQCT, robotic walking

## Abstract

**Introduction/Background:**

Bone and muscle health is a significant concern among individuals with neuromotor impairments, especially those with the greatest functional limitations. This case series presents the bone and muscle adaptations in response to 13 to 14 weeks of robotic walking in children and adolescents with neuromotor impairments.

**Methods:**

To ensure that the intervention targeted functionally meaningful outcomes, the Canadian Occupational Performance Measure (COPM) was used to capture family-centered goals and motivations for participating in robotic walking. Peripheral quantitative computed tomography (pQCT) scans of the tibia assessed bone and muscle health at three locations: the 3% (trabecular bone), 38% (cortical bone), and 66% (muscle) of tibia length. Scans were acquired before and following robotic walking in five participants with the greatest functional limitations [Gross Motor Function Classification System (GMFCS) levels III -V and Functional Assessment Questionnaire (FAQ): Functional Walking Scale levels 1 -5; aged 4 -12 years]. Bone and muscle outcomes were converted to age-, sex-, and ethnicity-matched *Z*-scores relative to a typically developing population, and the change in pre- and post-intervention *Z*-scores was assessed. The least significant change (LSC) was calculated for each participant in bone parameter units, and a clinically relevant change was defined as one that exceeded both the LSC and the expected growth from available reference data.

**Results:**

In some participants, clinically relevant improvements were observed in trabecular bone mineral density (BMD) (three participants), total bone mineral content (one participant), and cortical BMD (one participant) following robotic walking, suggesting promising bone adaptations.

**Conclusions:**

Results underscore the need for larger longitudinal studies to explore the impacts of robotic walking across various GMFCS and FAQ levels.

## 1 Introduction

Childhood and adolescence are critical periods for bone growth and development (1), making these years an important window to improve bone strength through physical activity, muscle–bone interactions, and gravitational forces (2). However, in neurodevelopmental disorders that impact physical function, like cerebral palsy (CP), bone health is often severely compromised (3–5). Children and adults with CP and other neuromotor impairments have smaller and thinner long bones (4, 6) and engage in significantly less physical activity than their typically developing peers (5, 7). Because of compromised musculoskeletal health, children and adults with CP are more prone to develop osteoporosis and experience fragility fractures (1, 8), resulting in pain and further declines in mobility. Targeted interventions during highly adaptive and critical periods, such as during childhood and adolescence, may be instrumental in improving bone and muscle strength and have the potential to reduce the declines in gross motor function known to increase with age (9).

In children with mobility impairments, their mobility is commonly characterized by two clinical scales: the Gross Motor Function Classification System (GMFCS) (10) and the Functional Assessment Questionnaire (FAQ): Functional Walking Scale (11). GMFCS categorizes functional abilities from walking without limitations (GMFCS I) to an inability to stand or propel oneself in a wheelchair (GMFCS V) (10). While GMFCS is the most commonly used scale, it is only validated for individuals with CP (12). The FAQ more specifically characterizes stepping and walking abilities in all children with mobility impairments (11). The FAQ is a family- or self-reported 10-level scale ranging from an inability to take any steps, even with support (FAQ 1), to walking household distances (>15–50 feet) inside at home or school (FAQ 5), and running and climbing comparable to typically developing peers (FAQ 10) (11). Youth with greater functional limitations participate in minimal weight-bearing activity, with most individuals with CP classified as GMFCS levels III–V achieving ≤30 min/day (13). Physical activity is essential for bone development and offers additional health benefits, such as reducing the risk of developing chronic diseases like diabetes, hypertension, cardiovascular conditions, joint pain, and arthritis (14, 15). For those with more severe functional impairments, recommendations include reducing sedentary time through standing and light physical activity (16). Therefore, interventions to reduce sedentary time and increase physical activity levels in children with mobility impairments are needed to improve health and potentially combat bone fragility.

While there are limited interventions to support physical activity in individuals with CP with the greatest functional limitations (GMFCS III–V), some target bone health, including vibration therapy, standing therapies, and body-weight-supported walking (4, 17–19). In these studies, concerns about the risk of fractures during treatment are common and often result in more conservative approaches. Additionally, participants find these therapy methods unengaging and tend to feel bored (19). Robotic walking has the potential to address several of these limitations. Recent advancements enable robot-assisted walking through multi-joint robotic devices that attach to participants’ limbs to support partial weight-bearing (20). Studies that evaluate robot-assisted walking aids in children and adolescents with CP commonly find that participants enjoy robotic walking (21), experience physical activity (22, 23), and have improvements in strength, gait speed, and gross motor function (21, 24, 25) following robotic walking. Despite these promising improvements, no studies have explored how this type of intervention impacts musculoskeletal health. Unlike volitional walking, robotic walking may permit forward progression with reduced muscle engagement. Because forward movement is generated through friction between the user’s foot and the floor, some lower-extremity loading is required even when body mass is partially supported by a seat. Therefore, robotic walking may provide mechanical stimuli relevant to musculoskeletal health, although the magnitude of these stimuli may differ from that experienced during volitional walking and remains underexplored. Furthermore, there is a gap between the general recommendations to promote bone health (e.g., calcium and vitamin D supplementation and weight-bearing exercise) and the use of screening, which is typically only implemented after a severe fracture has occurred, when consultation with a specialist and the consideration of bisphosphonates are often recommended (26). As a result, there is a gap in knowledge on how non-invasive proactive physical activity interventions can impact bone health.

This case series will address these limitations by assessing the impact of robotic walking on bone and muscle health with three-dimensional imaging in five children and adolescents. Specifically, this case series will examine whether 13–14 weeks of robotic walking influences bone density, geometry, and strength, as well as muscle size and density in children and adolescents with CP and other neuromotor impairments with the greatest functional limitations (GMFCS III–V and FAQ 1–5). We hypothesize that robotic walking will provide sufficient loading and physical activity from the introduction of upright mobility to improve tibial bone measures and muscle size and density.

## 2 Materials and methods

Participants from two ongoing clinical trials were invited to participate in this study. Diagnosis information, motivations, anthropometry, training plan, device usage logs, and bone and muscle imaging were collected on all participants. The primary purpose of both trials was to improve participant-specific goals relevant to their daily life. As both interventions involved dynamic weight-bearing activity in a population at risk of poor bone health, we decided to investigate the impacts on bone health. Study designs are described below. Written informed consent was obtained from the parents or guardians of all participants, and written/verbal assent was obtained from participants to participate in each respective study. Written informed consent was obtained from parents or guardians to be included in this case series. The requirement for updated informed consent was waived by the ethics board for one participant, as they had agreed to secondary data use when consenting to participate in the parent study. The study designs and case presentation were approved by the University of Calgary Conjoint Health Research Ethics Board (REB21-1166, REB24-0740, and REB26-0456).

### 2.1 Study designs

Participants from two clinical trials, titled Robotic Waking for Children Who Cannot Walk (RoWaCaWa) and Quantifying Unique Experiences in Single-participant Trials of robot-assisted walking (QUEST), were included in the study if they were at least 4 years of age, were unable to walk independently due to a childhood-onset neurological impairment, were able to fit the robotic walking aid (<150 lbs. and roughly <5′6″), and had no medical conditions that would preclude weight-bearing or physical activity. The RoWaCaWa trial is registered under www.clinicaltrials.gov as NCT05473676.

All participants included in this case series were lent a Trexo-Plus robotic walker (Trexo Robotics, Mississauga, ON, Canada; **Figure 1**) for 3 months of robotic walking. The robotic walker consists of a pair of robotic legs with motors aligned to the participant’s hips and knees attached to a Rifton Pacer walker for support and stability (27, 28) that moves the participant through a pre-set gait pattern. Participants are supported in the device with a removable and adjustable seat, which they can sit on if they are unable to support their whole body weight independently, as well as a chest strap and hand grips to hold on to if needed, allowing for partial to full weight-bearing. The device was fitted to each participant, and families or caregivers were trained to operate the robotic walker before training began. Participants in the RoWaCaWa trial were asked to use the device for ≥30 minutes per day for five or more days per week, over a 12-week duration. Participants in the QUEST study were instructed to use the robotic walker as intensively as possible, as determined by the family, but had no target goal in mind over 12 weeks. Although the interventions were 12 weeks long in both studies, participants had access to the walker after the intervention, and all participants used it at least once. Therefore, total robotic walking use between baseline and follow-up imaging was 13–14 weeks.

**Figure 1:**
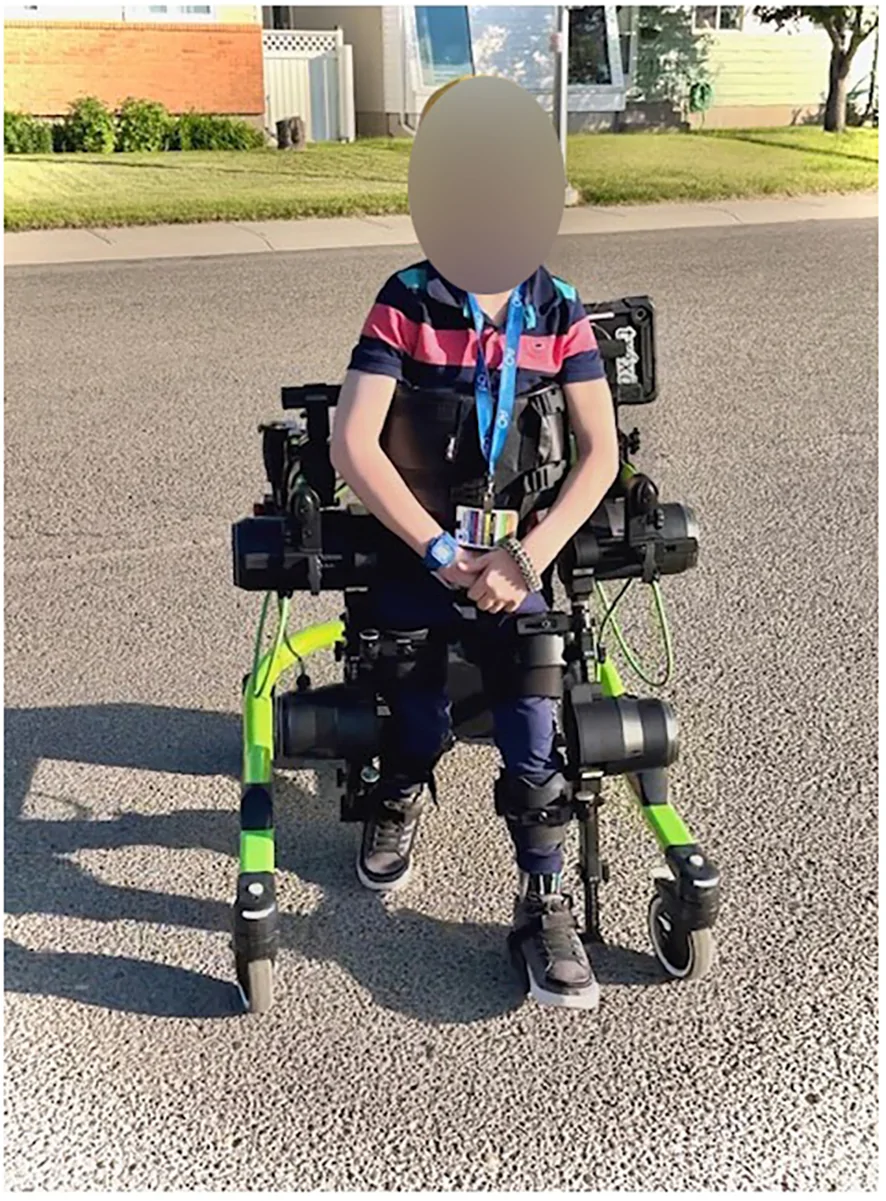
Participant walking in the Trexo Home robotic walking aid (Trexo Robotics).

### 2.2 Anthropometry

Tibia length was assessed to the nearest 0.1 cm as the distance from the medial malleolus to the tibial plateau (29), with measurements taken in duplicate if differences < 0.4 cm or triplicate if differences were >0.4 cm (29). The mean of two measurements or the median of three were used to calculate tibia length. Parents or caregivers reported the participants’ age, biological sex, diagnosis, and ethnicity in questionnaires. In QUEST, parents/caregivers also reported participation in other physical activities. Participants’ GMFCS levels were identified using the GMFCS Family Report Measure (10) and typical walking abilities with the FAQ: Functional Walking Scale (11). In RoWaCaWa and QUEST, height and weight were reported either by caregivers or through chart review. Heights were not available for all participants. Height and body mass index (BMI) *Z*-scores were calculated from the WHO Child Growth Standards (30).

### 2.3 Participant motivations

As the primary goal of both studies was to improve areas of function meaningful to participants and their families, the Canadian Occupational Performance Measure (COPM) (31) was used to capture and contextualize family-centered goals for participation. Clinicians guided parents through a conversation about their daily lives to determine goals that were important to the families and that had the potential to improve with robotic walking. COPM performance and satisfaction outcomes for the RoWaCaWa study have been previously reported (32). In this case series, COPM goal themes were used descriptively to characterize family motivations for participation and to determine whether caregivers perceived these goals to have been achieved following their intervention.

### 2.4 Bone and muscle imaging

Scans were acquired on both tibiae using the peripheral quantitative computed tomography (pQCT) XCT 3000 scanner (Stratec Medical, Pforzheim, Germany) by one of two trained operators as previously described (6). Bone imaging for RoWaCaWa occurred between September 2022 and February 2024, and between February 2025 and June 2025 in QUEST. In brief, all participants underwent pre-screening to ensure (a) no previous fracture/surgery or implanted metal in the lower limbs, (b) no medical devices that could be affected by the CT scanner, (c) no contrast imaging was administered within 5 days of the appointment, and (d) no chance of pregnancy at the time of the appointment. Adaptive seating, a calming environment in the scanner room including dimmed lights, a show or movie to watch, and stickers on the wall, as well as a video sent to families before the appointment, were used to increase scanning success (6).

Bone and muscle outcomes were acquired on both tibiae unless there was a previous relevant fracture or implanted metal in the lower limbs, in which case only the contralateral limb containing no fracture or metal was scanned. A daily and monthly hydroxyapatite cone phantom was scanned to maintain quality assurance. Based on 443 phantom scans, the long-term scanner coefficient of variation (CV, %) was 0.19% for trabecular attenuation (mean 0.448 ± 0.001 1/cm). A two-dimensional scout view was acquired, landmarking the growth plate in each participant, and a reference line was placed at the most distal aspect (33), with the resulting 3%, 38%, and 66% slices acquired proximal to this line. The manufacturer’s protocol was followed: 2.4 mm slice at 400 μm, a tube voltage of 46 kV DC, a current of <0.3 mA, with a scan speed of 30 mm/s. The scout view duration was ~80 s, the slice duration was ~100 s, and the total scan time was ~8 minutes. The effective dose was <3 μSV per series of scans. In instances of motion artifact, scans were repeated once. *In vivo* precision at each of these sites is excellent, 3% site: 3.0% (Tb.BMD) to 6.7% (Tt.BMC) (33); 38% site: 0.5% to 1.6% (Ct. BMD, Ct.BMC, Ct.Ar, Ct.Th, and endosteal circumference) (33–35) and 0.4% (periosteal circumference) (35); 66% site: <2% for muscle measurements (36).

A single researcher analyzed and evaluated all scans for motion artifact on a scale from 1 (no motion) to 5 (substantial motion) (37), with all scans with a grade of ≤3 at baseline prioritized at follow-up. All scans graded as 4 were reviewed by a second researcher to confirm exclusion. If a participant had no valid scans at baseline (grade of ≥4), they were not brought back for follow-up scans. If a participant had valid scans on the same leg at both baseline and follow-up (grading of ≤3), they were analyzed (Stratec software version 6.20). Analysis included one leg per participant. A manual ROI was drawn for all three sites (3%, 38%, and 66%). Analysis parameters for the 3% and 38% were peel and contour mode “1” with a trabecular area of 45%. The 3% measures used a threshold of 180 mg/cm^3^ and a cortical threshold of 480 mg/cm^3^, while the 38% bone measures used a threshold of 710 mg/cm^3^, and the strength measures used a threshold of 480 mg/cm^3^. The 66% slice muscle measures used a threshold of 280 mg/cm^3^ with contour mode “1”, peel mode “2”, and smoothing filter F0305. Muscle separation from subcutaneous fat utilized a threshold-based edge detection with a threshold of 40 mg/cm^3^ with contour mode “3”, peel mode “1”, and smoothing filter F030505. Resulting variables at the 3% slice include total bone mineral content (Tt.BMC; mg/mm) and trabecular bone mineral density (Tb.BMD; mg/cm^3^). Resulting variables at the 38% slice include cortical BMC (Ct.BMC; mg/mm), cortical BMD (Ct.BMD; mg/cm^3^), cortical thickness (Ct.Th; mm), cortical area (Ct.Ar; mm^2^), endosteal circumference and periosteal circumference (mm), section modulus (*Z*; mm^3^) and polar stress–strain index (SSI_p_; mm^3^), where *Z* is the bending resistance of cortical bone, while SSI_p_ is the density-weighted bending strength. Outcomes at the 66% slice include muscle cross-sectional area (MCSA; mm^2^) and muscle density (mg/cm^3^), where MCSA is the bone area subtracted from the total area and muscle density is the total muscle mass divided by MCSA. All values (excluding SSI_p_ and muscle density) were converted to age-, sex-, and ethnicity-matched *Z*-scores to a normative population (34). SSI_p_ and muscle density have no reference data in children.

### 2.5 Robotic walking usage

Robotic walking usage logs were downloaded from Amazon QuickSight (Amazon Web Services) following each training period. Data obtained included the start and end time, calculated duration, number of steps achieved, and number of starts. The mode (“strength” or “endurance”) of the device, and whether or not the removable seat was used, was noted by researchers or confirmed by Trexo. “Endurance mode” moves the participant through a pre-set gait pattern, while in “strength mode”, the pre-set gait pattern has a wider tolerance where the motors take longer to provide support when the gait deviates from the set trajectory.

### 2.6 Statistical analysis

Because of the limited samples in this case series and the inclusion of participants from QUEST who are known to have disorders that adversely impact skeletal aging, hypothesis testing and statistical testing were not performed. Instead, we descriptively assessed clinically relevant change in two ways. First, the change for each participant was compared to the least significant change (LSC) to determine whether it exceeded the scanner’s measurement error. Second, a more conservative approach assessed whether the change exceeded both the measurement error (LSC) and the expected change due to growth. Because longitudinal reference data describing expected bone adaptations in children and adolescents with neuromotor impairments are currently unavailable, expected growth was estimated using typically developing reference data. Although growth trajectories may differ between children and adolescents with neuromotor impairments and their typically developing peers, these data were used as a conservative benchmark rather than an estimate of participant-specific growth. The conservative LSC and expected growth threshold was selected to ensure that clinically relevant changes reflected values that would exceed growth alone and therefore increase our confidence that they were a result of the intervention. LSC was calculated as the CV previously reported (33–36) multiplied by 2.77 (38) and expressed in bone parameter units by multiplying the LSC (%) by the participants’ baseline measurement for each bone variable (39). To account for expected growth, the expected change for each bone parameter for each participant over the training period was calculated from normative data (e.g., the difference between the median reference data at each age over the intervention period) (34) and added to the LSC. Changes in bone values that exceeded both the LSC and expected growth were considered clinically relevant.

Usage metrics are presented as the median (interquartile range; 25th percentile to 75th percentile), unless indicated otherwise. These values were calculated over the entire duration of each participant’s training if >50 steps were achieved and the total session duration did not exceed 4 h, including their robotic walking education session(s), training period, and any extra training performed afterwards by the participant.

## 3 Results

This case series included five participants between the ages of 4 and 12 (median: 7.6). Three participants were male, and two were female. Two participants were diagnosed with CP, and three were diagnosed with rare genetic conditions. All participants identified as white or mixed ethnicity. Baseline descriptive characteristics are described in **Table 1**. A total of 276 robotic walking sessions were recorded across all participants.

**Table 1:** Baseline and follow-up values for Cases 1–5.

			RoWaCaWa									QUEST					
			Case 1—FAQ 2			Case 2—FAQ 5			Case 3—FAQ 3			Case 4—FAQ 3			Case 5—FAQ 1		
			Baseline	Follow-up	Change	Baseline	Follow-up	Change	Baseline	Follow-up	Change	Baseline	Follow-up	Change	Baseline	Follow-up	Change
	Demographics	Age (years)	4			6			9			7			12		
		Height (cm)	103.0	–	–	–	–	–	–	–	–	126.5	–	–	146.0	–	–
		BMI (kg/m^2^)	14.33	–	–	–	–	–	–	–	–	14.68	–	–	14.54	–	–
		BMI *Z*-score	−0.77	–	–	–	–	–	–	–	–	−0.54	–	–	−2.15	–	–
		GMFCS	IV			III			–			–			–		
	Tibia length	Tibia length (mm)	234	236	2.0	226	251	25.0	231	249	18.0	275	276	1.0	335	338	3.0
		Typically developing M	238.1	243.5	5.4	263.5	268.0	4.5	310.4	316.4	6.0	279.2	283.0	3.8	359.4	363.4	3.9
		*Z*-score	−0.31	−0.57	−0.26	−2.94	−1.20	1.74	−4.16	−3.51	0.65	−0.25	−0.41	−0.16	−1.25	−1.29	−0.04
3% Slice	Tb.BMD	Tb.BMD (mg/cm^3^)	110.7	121.1	10.4	133.1	159.9	26.8	221.2	222.4	1.2	125.4	166.7	41.3	–	–	–
		Typically developing M	224.3	225.0	0.7	227.6	228.1	0.6	228.3	228.9	0.6	227.3	227.1	−0.1	–	–	–
		*Z*-score	−5.94	−5.37	0.57	−4.67	−3.35	1.32	−0.31	−0.28	0.03	−4.81	−2.82	1.99	–	–	–
		LSC	–	–	9.2	–	–	11.1	–	–	18.4	–	–	10.4	–	–	–
		Clinically relevant	–	–	Yes	–	–	Yes	–	–	Growth	–	–	Yes	–	–	–
	Tt.BMC	Tt.BMC (mg/mm)	49.0	53.1	4.1	88.3	110.7	22.4	76.3	83.5	7.2	94.3	114.3	20.0	120.4	114.8	−5.6
		Typically developing M	115.1	120.4	5.4	138.8	142.6	3.8	170.0	177.0	7.0	138.3	141.3	3.0	249.2	255.0	5.8
		*Z*-score	−7.15	−6.81	0.34	−3.49	−1.88	1.61	−5.46	−5.13	0.33	−2.64	−1.47	1.18	−5.93	−6.60	−0.67
		LSC	–	–	9.1	–	–	16.4	–	–	14.2	–	–	17.5	–	–	22.4
		Clinically relevant	–	–	No	–	–	Yes	–	–	Growth	–	–	LSC	–	–	No
38% Slice	Ct.BMD	Ct.BMD (mg/cm^3^)	–	–	–	1,100.4	1,111.6	11.2	–	–	–	1,061.9	1,092.1	30.2	1,158.9	1,158.5	−0.4
		Typically developing M	–	–	–	1,054.2	1,054.8	0.5	–	–	–	1,050.7	1,051.2	0.5	1,075.4	1,077.3	1.8
		*Z*-score	–	–	–	1.34	1.72	0.38	–	–	–	0.37	1.43	1.06	3.30	3.21	−0.08
		LSC	–	–	–	–	–	15.2	–	–	–	–	–	14.7	–	–	16.1
		Clinically relevant	–	–	–	–	–	Growth	–	–	–	–	–	Yes	–	–	No
	Ct.BMC	Ct.BMC (mg/mm)	–	–	–	82.8	87.2	4.4	–	–	–	91.8	93.8	2.1	147.6	151.3	3.7
		Typically developing M	–	–	–	130.1	134.4	4.2	–	–	–	137.9	141.5	3.6	245.6	251.3	5.7
		*Z*-score	–	–	–	−3.67	−3.50	0.18	–	–	–	−2.85	−2.87	−0.02	−4.19	−4.18	0.01
		LSC	–	–	–	–	–	3.7	–	–	–	–	–	4.1	–	–	6.5
		Clinically relevant	–	–	–	–	–	Growth	–	–	–	–	–	No	–	–	No
	Ct.Ar	Ct.Ar (mm^2^)	–	–	–	75.2	78.4	3.2	–	–	–	86.4	85.9	−0.5	127.4	130.6	3.2
		Typically developing M	–	–	–	124.7	129.0	4.2	–	–	–	132.2	135.6	3.4	233.8	238.8	5.0
		*Z*-score	–	–	–	−4.06	−3.98	0.07	–	–	–	−3.07	−3.29	−0.22	−4.98	−4.95	0.03
		LSC	–	–	–	–	–	3.3	–	–	–	–	–	3.8	–	–	5.6
		Clinically relevant	–	–	–	–	–	No	–	–	–	–	–	No	–	–	No
	Ct.Th	Ct.Th (mm)	–	–	–	2.3	2.4	0.1	–	–	–	2.4	2.4	−0.0	3.1	3.1	0.0
		Typically developing M	–	–	–	3.4	3.5	0.1	–	–	–	3.4	3.5	0.1	4.7	4.7	0.1
		*Z*-score	–	–	–	−3.89	−3.79	0.10	–	–	–	−2.98	−3.18	−0.20	−3.47	−3.49	−0.02
		LSC	–	–	–	–	–	0.1	–	–	–	–	–	0.1	–	–	0.1
		Clinically relevant	–	–	–	–	–	No	–	–	–	–	–	No	–	–	No
	Endosteal circumference	Endosteal circumference (mm)	–	–	–	25.4	25.8	0.4	–	–	–	27.9	28.0	0.2	31.4	32.1	0.7
		Typically developing M	–	–	–	26.0	26.4	0.4	–	–	–	28.0	28.2	0.2	35.8	36.1	0.3
		*Z*-score	–	–	–	−0.14	−0.16	−0.02	–	–	–	−0.04	−0.05	−0.01	−0.94	−0.84	0.10
		LSC	–	–	–	–	–	1.1	–	–	–	–	–	1.2	–	–	1.4
		Clinically relevant	–	–	–	–	–	Growth	–	–	–	–	–	Growth	–	–	Growth
	Periosteal circumference	Periosteal circumference (mm)	–	–	–	39.9	40.6	0.7	–	–	–	43.1	43.2	0.1	50.8	51.7	0.8
		Typically developing M	–	–	–	47.4	48.1	0.8	–	–	–	49.5	50.1	0.6	65.2	65.8	0.7
		*Z*-score	–	–	–	−2.39	−2.36	0.03	–	–	–	−1.99	−2.13	−0.14	−3.49	−3.40	0.09
		LSC	–	–	–	–	–	0.4	–	–	–	–	–	0.5	–	–	0.6
		Clinically relevant	–	–	–	–	–	LSC	–	–	–	–	–	No	–	–	LSC
	*Z*	Z (mm^3^)	–	–	–	271.7	298.2	26.5	–	–	–	356.8	354.4	−2.4	578.5	604.7	26.2
		Typically developing M	–	–	–	531.7	557.6	26.0	–	–	–	591.6	612.1	20.5	1,333.2	1,373.6	40.4
		*Z*-score	–	–	–	−3.08	−2.92	0.16	–	–	–	−2.59	−2.79	−0.21	−5.41	−5.31	0.11
		LSC	–	–	–	–	–	12.0	–	–	–	–	–	15.8	–	–	25.6
		Clinically relevant	–	–	–	–	–	LSC	–	–	–	–	–	No	–	–	LSC
	SSI_p_	SSI_p_ (mm^3^)	–	–	–	367.9	395.3	27.4	–	–	–	491.9	488.9	−3.0	748.5	791.2	42.7
66% Slice	MCSA	MCSA (mm^2^)	1,227.8	1,284.2	56.3	–	–	–	–	–	–	1,579.8	1,701.9	122.1	2,061.4	2,125.0	63.5
		Typically developing M	2,478.8	2,596.8	118.0	–	–	–	–	–	–	3,146.6	3,205.0	58.4	5,230.2	5,361.5	131.3
		*Z*-score	−4.20	−4.21	−0.01	–	–	–	–	–	–	−4.07	−3.76	0.31	−5.37	−5.34	0.03
		LSC	–	–	68.0	–	–	–	–	–	–	–	–	87.5	–	–	114.2
		Clinically relevant	–	–	No	–	–	–	–	–	–	–	–	LSC	–	–	No
	Muscle density	Muscle density (mg/cm^3^)	74.0	76.1	2.1	–	–	–	–	–	–	75.7	74.8	−0.9	75.8	75.9	0.1

M, median; LSC, least significant change; BMI, body mass index; Tb.BMD, trabecular bone mineral density; Tt.BMC, total bone mineral content; Ct.BMD, cortical bone mineral density; Ct.BMC, cortical bone mineral content; Ct.Ar, cortical area; Ct.Th, cortical thickness; *Z*, section modulus; SSI_p_, polar stress–strain index; MCSA, muscle cross-sectional area. Yes (green) indicates that change exceeds the LSC and growth. LSC (orange) indicates that the change exceeds the LSC. Growth (yellow) indicates that the change matches or exceeds the expected growth in typically developing reference data. No indicates that change is not significant at any threshold.

### 3.1 Case 1

Case 1 is a 4-year-old boy diagnosed with bilateral mixed (spastic and dyskinetic) CP, GMFCS level IV, with a family-reported FAQ of 2. The family’s motivations for participating in robotic walking were to improve independent mobility within their gait aid, bowel function, and sleep, and to feel greater inclusion with peers.

This participant was taking laxatives throughout the study and baclofen at the beginning of the study, which was weaned over the first 7 weeks. This participant was younger than the available reference data; therefore, data were compared to reference data 5.1 years of age.

#### 3.1.1 Usage

Case 1 used the robotic walker with the seat in “endurance mode” for 58 sessions, totaling 1,795 minutes and 100,353 steps. They used the device for a median of 4 sessions per week (range: 2–7). Each session lasted for a median of 32 (27–34) minutes, achieving a median of 1,828 (1,449–2,062) steps.

#### 3.1.2 Results

Following the training period, the family noted improvements in independent motion in their walker, better upright walking without adult support, and a greater sense of inclusion while walking with peers. Caregivers reported a decline in bowel movement frequency and sleep duration over the intervention.

Their right limb had better scans and was included in the analysis (**Figure 2**). Bone outcomes were significantly lower than typically developing reference data at baseline, with Tb.BMD and Tt.BMC *Z*-scores of −5.94 and −7.15, respectively, at the 3% site. Following robotic walking, both Tb.BMD and Tt.BMC *Z*-scores improved by 0.57 and 0.34, respectively. Improvements in Tb.BMD raw values (+10.4 mg/cm^3^) exceeded the LSC (+9.2 mg/cm^3^) and LSC and expected growth for the typically developing reference data over the 3 months (+9.9 mg/cm^3^), representing a clinically relevant improvement. Improvements in raw values for Tt.BMC (+4.1 mg/mm) did not exceed the LSC (+9.1 mg/mm) or the LSC and expected growth (+14.5 mg/mm).

**Figure 2:**
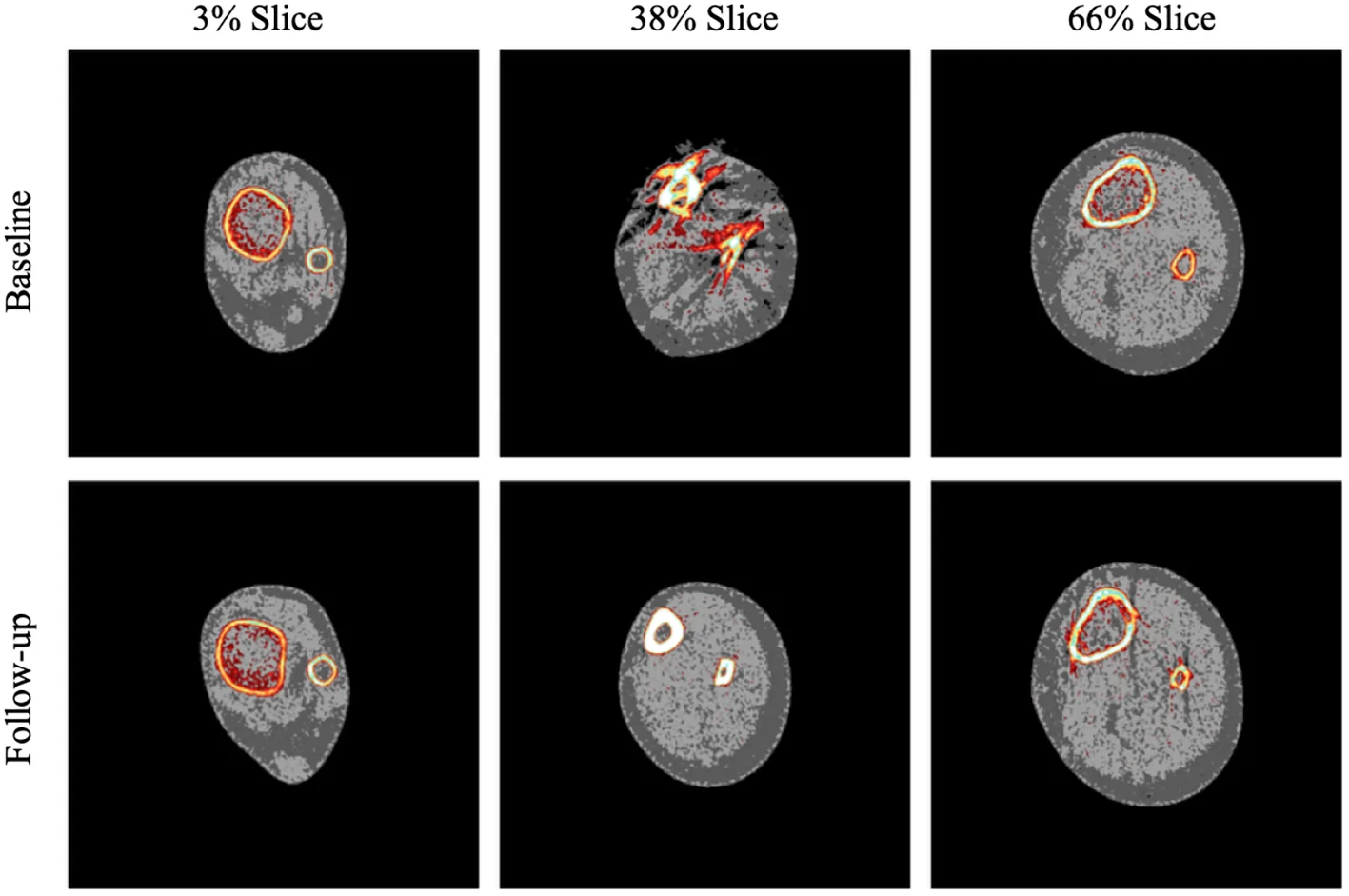
Baseline and follow-up peripheral quantitative computed tomography (pQCT) scans from Case 1’s right limb at the 3%, 38% and 66% slices. Note: The baseline 38% slice has significant motion and is not included in the analysis.

Due to significant motion in the scans at the 38% baseline slice, no data were analyzed.

Muscle measures at the 66% slice showed a significantly lower MCSA *Z*-score than typically developing reference data (−4.20), which did not change (−0.01) over the intervention. Changes in the MCSA (+56.3 mm^2^) did not exceed the LSC (+68.0 mm^2^) or the LSC and growth (+186.0 mm^2^). Muscle density increased from 74.0 mg/cm^3^ to 76.1 mg/cm^3^ over the intervention.

### 3.2 Case 2

Case 2 is a 6-year-old boy diagnosed with bilateral spastic CP, GMFCS level III, with a family-reported FAQ of 5. This participant’s motivations for robotic walking were to walk with a smoother, more defined gait and to play with their sibling in the walker. They were not taking any medication that may have impacted bone during this trial.

#### 3.2.1 Usage

Case 2 utilized the robotic walker with the seat in “endurance mode” for the first 4 weeks of their training, then continued using the seat and used “strength mode” for the remaining 8 weeks of the intervention. They completed a total of 72 sessions, totaling 3,334 minutes and 171,437 steps. They used the device for a median of 6 sessions per week (range: 1–8). Each session lasted for a median of 49 (42–53) minutes, achieving a median of 2,665 (2,188–2,785) steps.

#### 3.2.2 Results

Following the training period, the family noted that they were able to walk with a better gait pattern and play with their sibling while in the walker.

Their right limb had better scans and was included in the analysis (**Figure 3**). Bone outcomes were significantly lower than typically developing reference data at baseline, with Tb.BMD and Tt.BMC *Z*-scores of −4.67 and −3.49, respectively, at the 3% site. Following robotic walking, both Tb.BMD and Tt.BMC *Z*-scores improved by 1.32 and 1.61, respectively. Improvements in Tb.BMD raw values (+26.8 mg/cm^3^) exceeded the LSC (+11.1 mg/cm^3^) and LSC and expected growth for the typically developing reference data over the 3 months (+11.7 mg/cm^3^), which were visually apparent across scans (**Figure 3**), representing a clinically relevant improvement. Improvements in raw values for Tt.BMC (+22.4 mg/mm) exceeded the LSC (+16.4 mg/mm) and LSC and expected growth (+20.2 mg/mm), representing a clinically relevant improvement.

**Figure 3:**
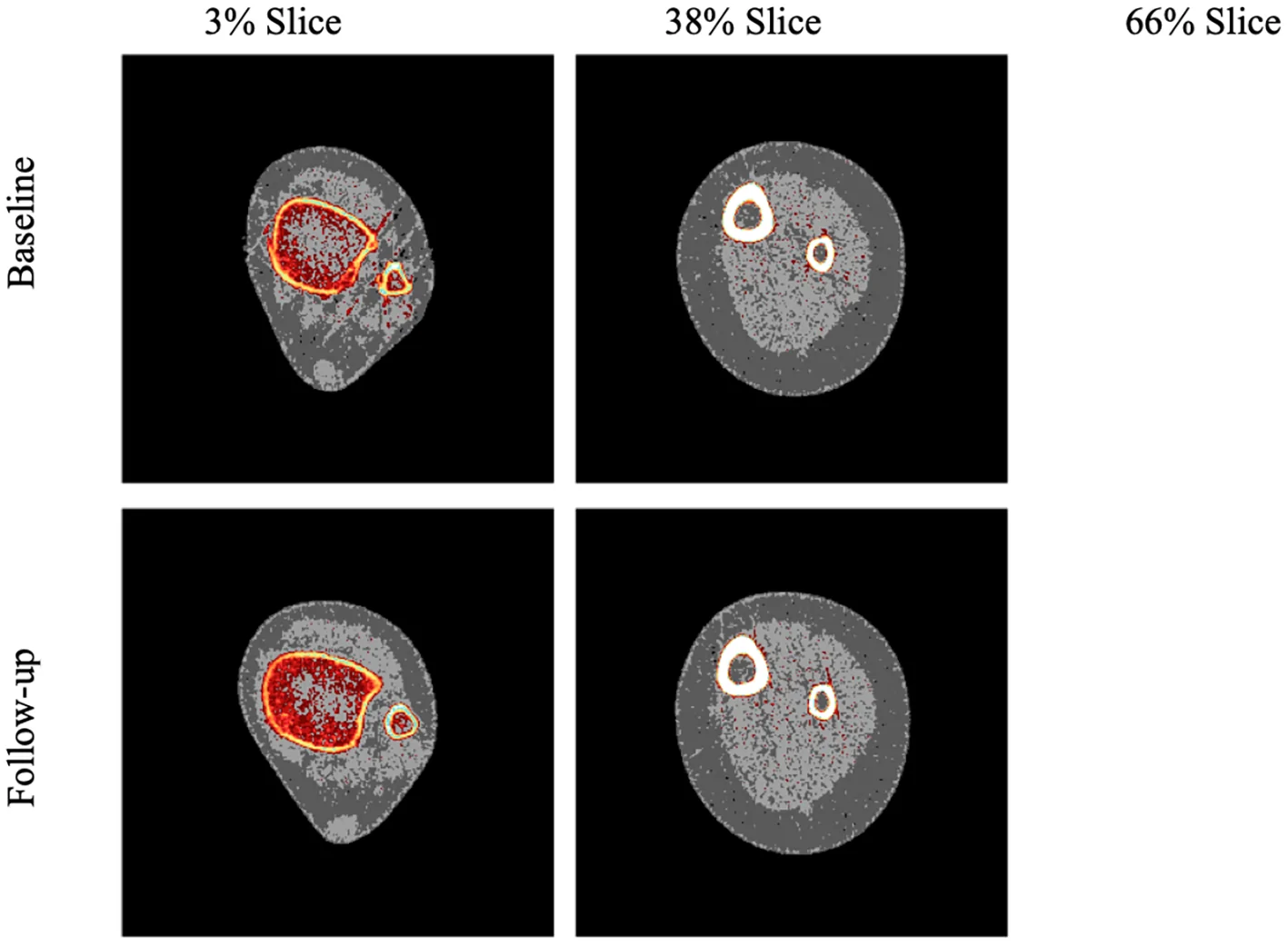
Baseline and follow-up peripheral quantitative computed tomography (pQCT) scans from Case 2’s right limb at the 3% and 38% slices. Note: No 66% slices were obtained.

At the 38% slice, Ct.BMD was higher than typically developing reference data with a *Z*-score of 1.34 at baseline, which increased by 0.38 during the intervention. Raw change in Ct.BMD (+11.2 mg/cm^3^) exceeded those expected due to growth in a typically developing population, but did not exceed the LSC (+15.2 mg/cm^3^) or the LSC and expected growth (+15.7 mg/cm^3^). Ct.BMC, Ct.Th, Ct.Ar, periosteal circumference, and section modulus were lower than a typically developing reference population at baseline, with *Z*-scores ranging from −4.06 to −2.39, which maintained or improved following 12 weeks of robotic walking (*Z*-score improvements ranged from 0.03 to 0.72). Ct.Ar improved over the intervention but was 0.1 mm^2^ shy of meeting the LSC threshold. Endosteal circumference *Z*-score was normal at baseline (−0.14) and did not change (−0.01) over the intervention. As Ct.BMD and endosteal circumference *Z*-scores were within a healthy range at baseline, large improvements may be less likely over the intervention. Changes in Ct.BMC (+4.4 mg/mm), periosteal circumference (+0.7 mm), and section modulus (+26.5 mm^3^) exceeded the LSC, but none exceeded the LSC and expected growth (+7.9 mg/mm; +1.2 mm; +38.0 mm^3^, respectively). Changes in endosteal circumference over the intervention (+0.4 mm) matched the expected growth in a typically developing population but did not exceed the LSC (+1.1 mm) or LSC and growth (+1.15 mm). SSI_p_ was 367.9 mm^3^ at baseline and increased by 27.4 mm^3^ over the intervention.

No scans were acquired at the 66% slice.

Tibial length increased by 25 mm over the intervention period, compared with a 16 mm increase in the contralateral limb. Although this suggests a possible overestimation of limb length at the follow-up visit, the use of relative scan locations (3% and 38% of limb length) means this discrepancy would shift the scan site proximally by only 0.27 mm. Given the small magnitude of this shift relative to slice thickness and regional variation in bone measures, any effect on the derived outcomes is expected to be negligible. Accordingly, these scans were included in the final analyses.

### 3.3 Case 3

Case study 3 is a 9-year-old girl diagnosed with a non-progressive genetic disorder causing bilateral motor impairments with a family-reported FAQ of 3. This condition does not include endocrine abnormalities known to influence bone. This participant was fed via a G-tube as guided by their dietitian before and throughout this intervention and was not on any medications known to impact bone. The family’s motivations for participating in robotic walking were to increase the frequency of steps taken in their walker and their ability to stand with support, increase their ability to reach and grasp items, and increase their sleep duration.

#### 3.3.1 Usage

Case 3 used the robotic walker without the seat and used “endurance mode” throughout their training. They completed a total of 63 sessions, totaling 3,658 minutes and 102,748 steps. They used the device for a median of 5 sessions per week (range: 1–8). Each session lasted for a median of 50 (38–60) minutes, achieving a median of 1,562 (1,274–1,965) steps.

#### 3.3.2 Results

Following the training period, the family noted improved frequency of their steps in the walker, maintenance of their head control, increased duration of standing with support, and greater ability to reach and grab items. Sleep performance and duration were satisfactory before and after training.

Their left limb had better scans and was included in the analysis (**Figure 4**). Case 3 had a normal Tb.BMD *Z*-score of −0.31, which is visible in scans (**Figure 4**), but a significantly lower Tt.BMC *Z*-score of −5.46 at the 3% slice. Following robotic walking, both Tb.BMD and Tt.BMC *Z*-scores were maintained or improved by 0.03 and 0.33, respectively. Improvements in Tb.BMD raw values (+1.2 mg/cm^3^) exceeded the expected growth for the typically developing reference data (0.6 mg/cm^3^) but did not exceed the LSC (+18.4 mg/cm^3^) or LSC and expected growth (+19.0 mg/cm^3^). As trabecular bone was within a healthy range at baseline, large improvements may be less likely. Improvements in raw values for Tt.BMC (+7.2 mg/mm) exceeded expected growth (+7.0 mg/mm) but did not exceed the LSC (+14.2 mg/mm) or the LSC and expected growth (+21.2 mg/mm).

**Figure 4:**
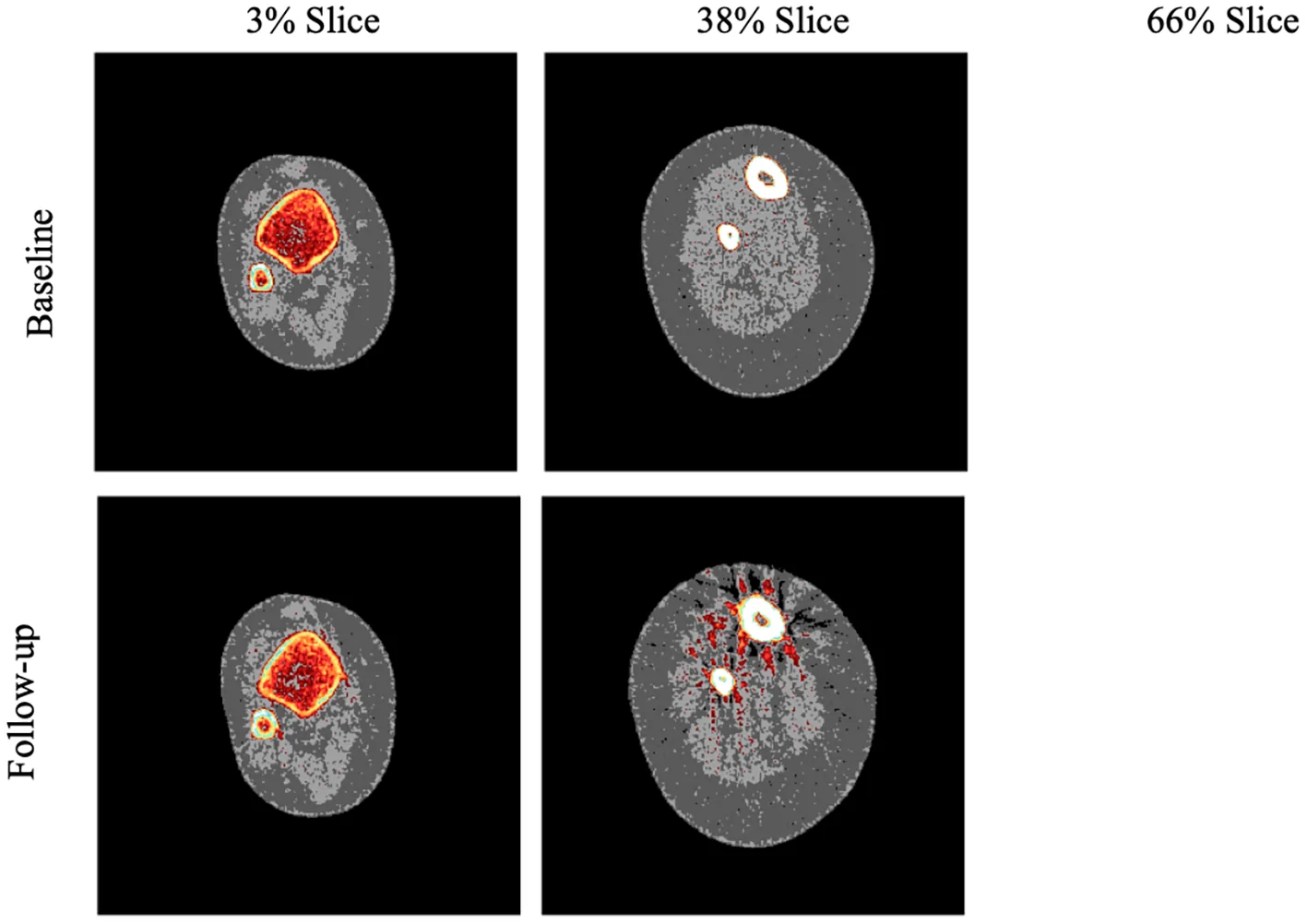
Baseline and follow-up peripheral quantitative computed tomography (pQCT) scans from Case 3’s left limb at the 3% and 38% slices. Note: No 66% scans were obtained.

Because of significant motion in the follow-up 38% slice, data at that site were not analyzed. No scans were acquired at the 66% slice.

### 3.4 Case 4

Case 4 is a 7-year-old girl diagnosed with a genetic disorder causing bilateral motor impairments with a family-reported FAQ of 3, whose condition can include endocrine anomalies such as precocious puberty and advanced bone age. Family motivations to participate in robotic walking were to improve leg strength, postural control, bowel function, and sleep, and to enjoy new community experiences.

This participant was on numerous medications while participating in training, including lupron, epidiolex, ethosuximide, vigabatrin, lorazepam, metoclopramide, and naproxen as needed. While these medications may have an impact on bone, they were taken consistently before and throughout the intervention, and therefore, we do not expect them to explain any changes in bone observed in this study. This participant stood in a standing frame, used a walker, and used a therapeutic pool while at school during the training period.

#### 3.4.1 Usage

Case 4 used the robotic walker with the seat and used “endurance mode” throughout their training. They completed a total of 46 sessions totaling 1,655 minutes and 33,989 steps in the robotic walker. They used the device for a median of 4 sessions per week (range: 1–5). Each session lasted for a median of 34 (28–53) minutes, achieving a median of 773 (519–992) steps.

#### 3.4.2 Results

Following the training period, the family noted improvements across all motivation areas, including increased leg strength and postural control, greater participation in new community experiences, and, especially, improvements in bowel movement frequency and sleep duration.

Their right limb had better quality scans and was included in the analysis (**Figure 5**). Bone outcomes were significantly lower than typically developing reference data at baseline, with Tb.BMD and Tt.BMC *Z*-scores of −4.81 and −2.64, respectively, at the 3% site. Following robotic walking, both Tb.BMD and Tt.BMC *Z*-scores improved by 1.99 and 1.18, respectively. Improvements in Tb.BMD raw values (+41.3 mg/cm^3^) were visually apparent (**Figure 5**), exceeding the LSC (+10.4 mg/cm^3^) and the LSC and expected growth for the typically developing reference data over the 3 months (+10.3 mg/cm^3^). Improvements in raw values for Tt.BMC (+20.0 mg/mm) also exceeded the LSC (+17.5 mg/mm) and the expected growth (+3.0 mg/mm), but was 0.5 mg/mm below the conservative LSC and expected growth (+20.5 mg/mm) threshold.

**Figure 5:**
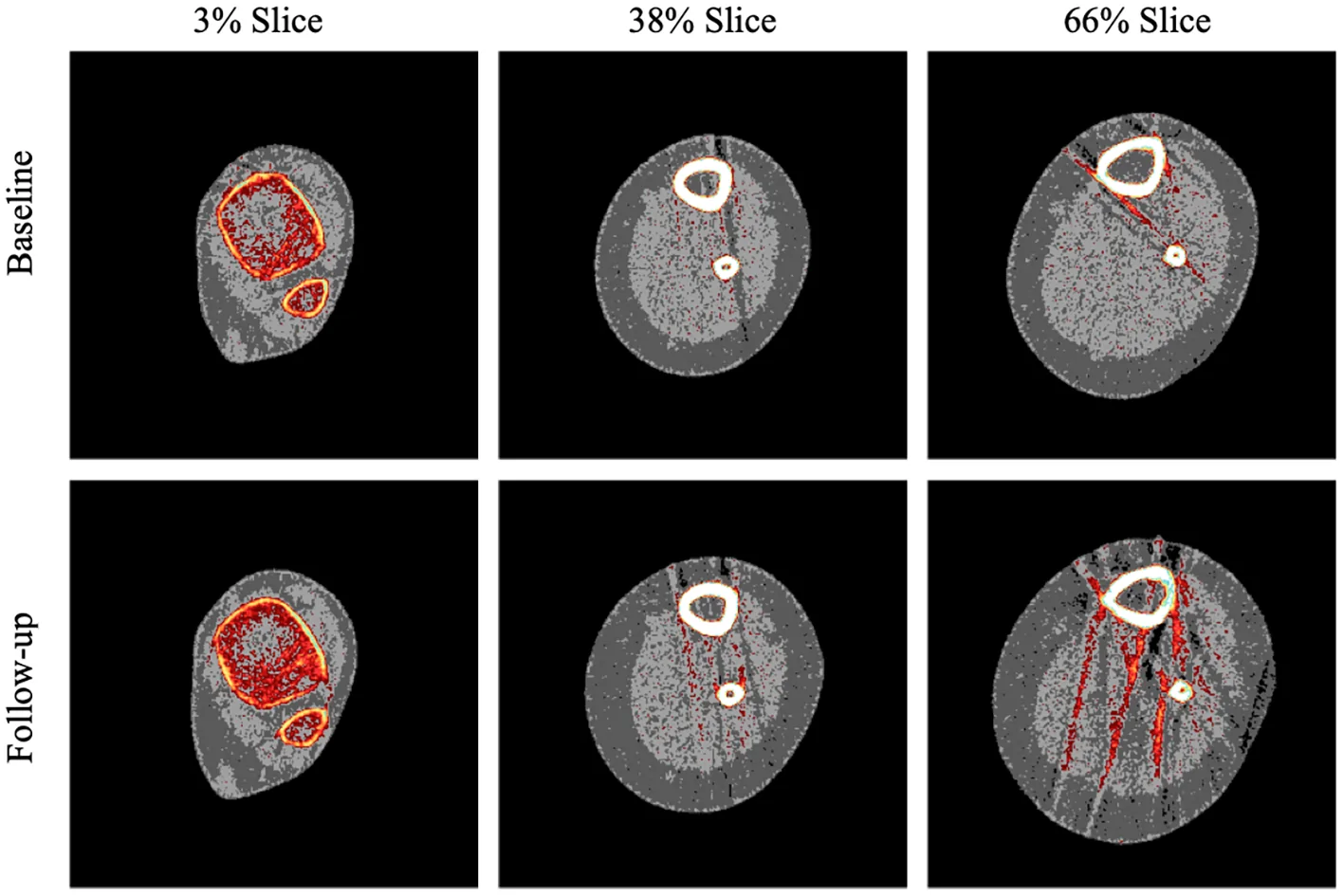
Baseline and follow-up peripheral quantitative computed tomography (pQCT) scans from Case 4’s right limb at the 3%, 38%, and 66% slices.

At the 38% site, Ct.BMD was greater than expected from typically developing reference data with a *Z*-score of 0.37 at baseline, which increased by 1.06 following the intervention. Similarly, the Ct.BMD raw value improved over the intervention (+30.2 mg/cm^3^), exceeding the LSC (+14.7 mg/cm^3^) and the LSC and expected growth (+15.2 mg/cm^3^). The *Z*-scores for Ct.BMC, Ct.Th, Ct.Ar, periosteal circumference, and section modulus were lower than the typically developing reference population, with *Z*-scores ranging from −3.07 to −1.99, all of which were maintained (changes ranged from −0.22 to −0.02) over the intervention. Endosteal circumference *Z*-score was normal at baseline (−0.04) and did not change (−0.01) over the intervention. SSI_p_ was 491.9 mm^3^ at baseline and decreased by 3.0 mm^3^ over the intervention. The raw values for Ct.BMC, endosteal circumference, and periosteal circumference improved over the intervention (+2.1 mg/mm, +0.2 mm, and +0.1 mm, respectively) but did not exceed their respective LSC values (+4.1 mg/mm, +1.2 mm, and +0.5 mm, respectively). Changes in endosteal circumference over the intervention (+0.2 mm) matched the expected growth in a typically developing population.

MCSA *Z*-score at the 66% slice was significantly lower than typically developing reference data (−4.07), which improved by 0.31 over the intervention. Changes in the MCSA (+122.1 mm^2^) exceeded the LSC (+87.5 mm^2^) and the expected growth (+58.4 mm^2^), but not the LSC and expected growth (+145.9 mm^2^). Muscle density decreased from 75.7 to 74.8 mg/cm^3^ over the intervention.

### 3.5 Case 5

Case 5 is a 12-year-old boy diagnosed with a presumed genetic condition causing bilateral neuromotor impairments with a family-reported FAQ of 1. Family motivations to participate in robotic walking were to improve their bowel function, ability to stretch their legs without adult support, increase their walking ability in school and with family, and their sense of inclusion with peers.

This participant was on the following medications during their intervention: tizanidine, lorazepam, levodopa-carbidopa, and eslicarbazepine. While these medications may have an impact on bone, they were taken consistently before and throughout the intervention, and therefore, we do not expect them to explain any changes in bone observed in this study. This participant also participated in supportive standing, physiotherapy, and power hockey over the training period.

#### 3.5.1 Usage

Case 5 used the robotic walker without a seat and used “endurance mode” throughout training. They completed a total of 37 sessions, totaling 1,457 minutes and 37,440 steps. They used the device for a median of 2 sessions a week (range: 1–7). Each session lasted for a median of 37 (31–49) minutes, achieving a median of 1,011 (563–1,385) steps.

#### 3.5.2 Results

Following their training, the family noted improvements in all outlined goals, including their ability to stretch their legs without adult assistance, their walking ability in school and with family, a greater sense of inclusion with peers, and an increased frequency of bowel movements.

Their right limb had better scans and was included in the analysis (**Figure 6**); however, due to motion at the 3% slice, only Tt.BMC was assessed. Tt.BMC was significantly lower than typically developing reference data at baseline, with a *Z*-score of −5.93. Following robotic walking, the Tt.BMC *Z*-score declined by 0.67. Change in raw Tt.BMC (−5.6 mg/mm) did not exceed the LSC (+22.4 mg/mm).

**Figure 6:**
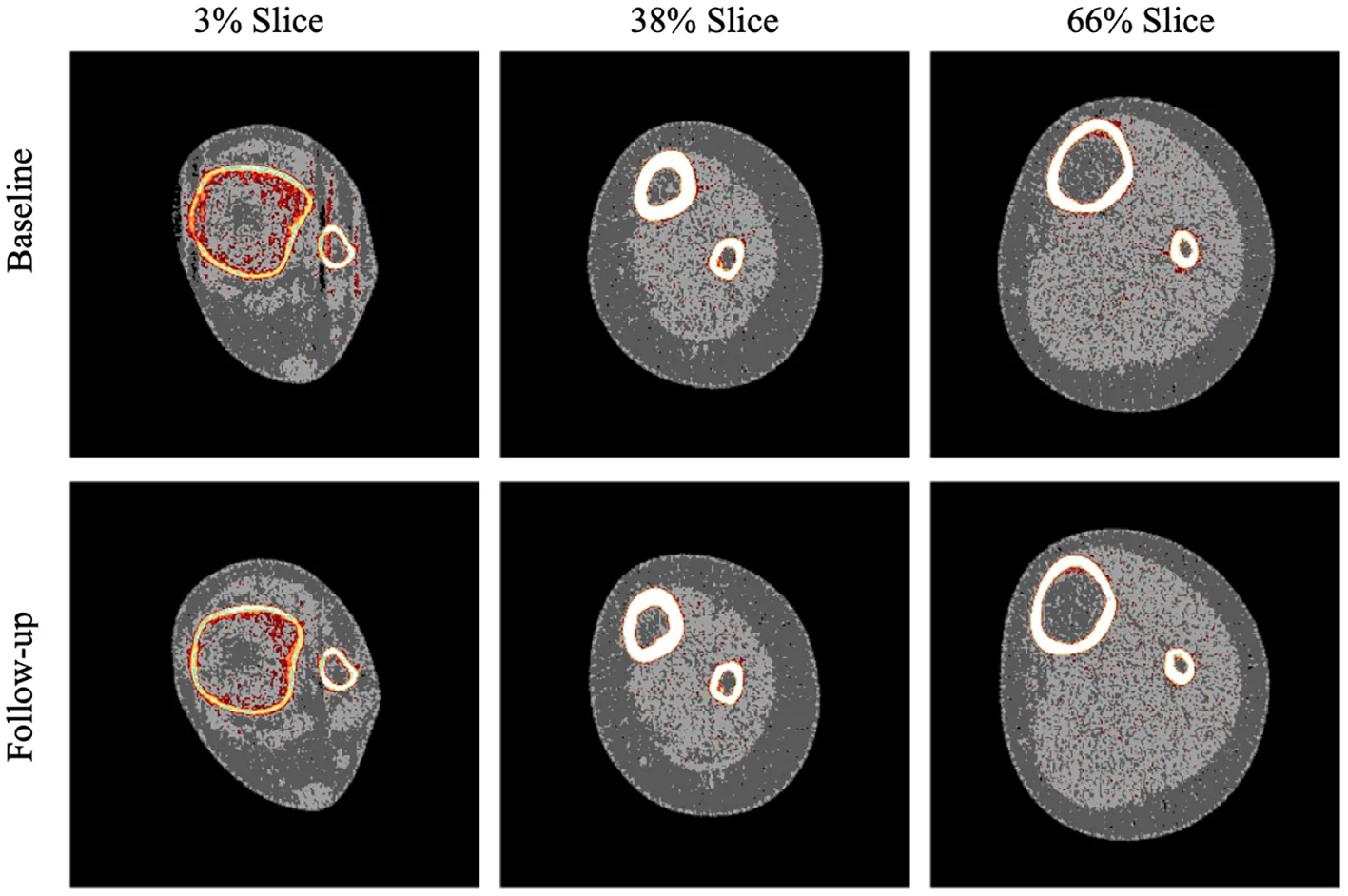
Baseline and follow-up peripheral quantitative computed tomography (pQCT) scans from Case 5’s right limb at the 3%, 38% and 66% slices.

Ct.BMD from the 38% slice was higher than the typically developing reference data, with a *Z*-score of 3.30 at baseline, which declined by 0.08 over the intervention. The raw density of Ct.BMD did not change over the intervention (−0.4 mg/cm^3^). The *Z*-scores for Ct.BMC, Ct.Th, Ct.Ar, periosteal circumference, and section modulus were lower than those of a typically developing reference population, with *Z*-scores ranging from −5.41 to −3.47, and all were maintained or increased by −0.02 to +0.11 over the intervention. Endosteal circumference *Z*-score was normal at baseline (−0.94) and increased by 0.10 over the intervention, with a raw change (+0.7 mm) exceeding those expected in the typically developing reference data but not the LSC or LSC and growth. The raw values for Ct.BMC, Ct.Th, Ct.Ar, periosteal circumference, and *Z* were maintained or improved over the intervention (+3.7 mg/mm, +0.0 mm, +3.2 mm^2^, +0.8 mm, and +26.2 mm^3^), where periosteal circumference and section modulus exceeded the LSC (+0.6 mm and +25.6 mm^3^, respectively) but not the LSC and expected growth (+1.3 mm and +66.0 mm^3^, respectively). SSI_p_ was 748.5 mm^3^ at baseline and improved by +42.7 mm^3^ over the intervention.

Muscle measures at the 66% slice showed a significantly lower MCSA *Z*-score than typically developing reference data (−5.37), which did not change (+0.03) over the intervention. Changes in MCSA (+63.5 mm^2^) did not exceed the LSC (+114.2 mm^2^) or the LSC and expected growth (+245.5 mm^2^). Muscle density was maintained over the intervention (+0.1 mg/cm^3^).

## 4 Discussion

This case series explored changes in bone and muscle outcomes following 3 months of robotic walking in children and adolescents with neuromotor impairments with a wide range of ages, mobility levels, and clinical characteristics. Interpreting skeletal adaptations during childhood and adolescence is inherently complex as these periods are characterized by transient, non-linear growth with rapid changes in bone geometry, mineral accrual, and muscle development that vary by age, pubertal status, and mechanical loading (40–42). Furthermore, growth trajectories in individuals with CP and other neuromotor impairments are challenging to characterize and can vary substantially between individuals. Therefore, presentation of case-specific assessments relative to known reference values in typically developing youth, scanner-specific measurement error, and conservative thresholds may provide insights to differentiate growth from intervention effects. While conclusions are difficult to draw due to the diversity in age, diagnosis, functional ability, and usage, trends from this work have the potential to guide future research assessing the influence of robotic walking on bone health.

At baseline, visual deficits in Tb.BMD and Tt.BMC at the distal tibia were observed in nearly all participants. With the exception of one participant whose Tb.BMD *Z*-score fell within the expected range at baseline, all participants demonstrated substantially reduced Tb.BMD and Tt.BMC, which is consistent with previous studies assessing bone health in children with CP and other neuromotor impairments (6, 43). Following robotic walking, improvements in Tb.BMD exceeded our conservative, clinically relevant thresholds in three of the four participants, with changes consistent with or exceeding the expected growth trajectories in the normative reference data for all four participants. These changes were visually apparent in the distal metaphysis pQCT scans (**Figures 3** and **5**), suggesting that robotic walking over a short 3-month duration may have a greater influence on the more metabolically active trabecular bone tissue in the distal tibia in those with baseline deficits.

At the diaphysis, baseline Ct.BMD and endosteal circumference *Z*-scores were above or within the expected range of the typically developing reference data, which is consistent with prior work that noted no differences in Ct.BMD between two sex- and age-matched typically developing controls (4). This also aligns with our prior work comparing a larger cohort of adolescents with CP to typically developing normative data, in which children and adolescents with CP had thinner long bones but appropriate cortical mineralization (6). While modest increases in Ct.BMD and endosteal circumference were observed following the intervention; only one participant’s change in Ct.BMD exceeded our conservative clinical threshold. Because Ct.BMD and endosteal circumference *Z*-scores were within normal ranges at baseline, we would not have expected changes to exceed the conservative clinically relevant thresholds. Despite this, one participant demonstrated Ct.BMD gains substantially greater than those expected from growth alone, a second participant exceeded the conservative clinically relevant threshold, and all three participants met or exceeded what was expected due to growth in endosteal circumference. The third participant showed no change in Ct.BMD, which may reflect normal individual variation in growth and skeletal adaptation. In contrast, other cortical content and geometry measures remained below typically developing reference values and demonstrated minimal change. While robotic walking requires some loading through the friction between the user and the ground, the degree of loading is unknown. Therefore, the limited improvement in cortical measures may reflect the study duration being insufficient to observe cortical modeling, how loading is experienced along the bone, or the influence of using a seat. Future work is needed to quantify the loading experienced while robotic walking, explore how the “strength” or “endurance” modes affect muscle activation, and assess the influence of the seat.

MCSA was substantially lower at baseline and showed limited responsiveness to the intervention, with only one participant demonstrating changes that exceeded the expected growth and measurement error independently. This may reflect characteristics of robotic walking, in which the foot is secured in a footplate, and the limb is guided through a pre-set gait pattern, not requiring active muscle engagement while walking. Additionally, most participants commonly use ankle-foot orthoses in daily life and would have continued wearing them during their robotic walking, which may further restrict ankle motion and calf muscle activation.

Interpretations of the trends presented herein reflect a longitudinal snapshot between two timepoints, acknowledging that there is substantial inter-individual variability across growth in children and adolescents with neuromotor impairments. Our results do not account for pubertal status, concurrent therapies, or other factors that may influence bone, such as medication exposure or nutritional status.

Despite these limitations, these results suggest that robotic walking may elicit compartment-specific skeletal adaptations, with more consistent improvements observed in Tb.BMD in those with deficits at baseline compared with those with normal values or those observed in cortical bone following 3 months of robotic walking. These findings may have clinical relevance, given that lower bone density and strength have been associated with greater fracture risk during childhood (44, 45). In children, each SD decrease in pQCT and dual-energy X-ray absorptiometry (DXA)-derived bone measures has been associated with 28%–41% greater odds of forearm fracture (44), while fracture risk increased by 89% for every SD decrease in size-adjusted DXA-derived BMC (45). Therefore, interventions that improve bone health during growth may have the potential to reduce skeletal fragility. This case series provides preliminary evidence to support further investigation of robotic walking to address musculoskeletal fragility during highly adaptive periods of growth in children and adolescents with neuromotor impairments to optimize bone health.

## 5 Limitations

This research has several limitations. Due to the difficulty of acquiring scans in this population, our sample size was small, captured over a short duration, and participants varied widely in age, diagnosis, family-reported mobility level, baseline musculoskeletal health, concurrent interventions, medication exposure, and device usage characteristics. Similarly, as this study was aimed at improving aspects of daily life, factors that could influence bone, such as nutrition levels, dietary calcium, vitamin D intake, hormone levels, or biochemical markers of bone turnover, were not assessed. Furthermore, the clinically relevant threshold was conservative and derived from population-level typically developing reference data. As we cannot differentiate between intervention effects and natural growth, the clinically relevant threshold was conservative and may not fully capture individual variability expected in children with neuromotor impairments. Future work would benefit from the inclusion of a control group. Finally, many outcomes were assessed for each participant; therefore, while the conservative threshold was intended to reduce false-positive classifications, they remain possible. Despite these limitations, this case series adds to the body of evidence on musculoskeletal adaptations in youth with neuromotor impairments and provides preliminary data to inform the design of future studies.

## 6 Conclusions

Collectively, these findings suggest that robotic walking may benefit musculoskeletal health in some children, particularly at the metaphyseal site. However, the variability in responses emphasizes the need for larger, longitudinal studies with a longer assessment period, a larger sample size across all functional levels and ages, and greater exploration of robotic walking parameters, such as dose, seat usage, and mode. Future work assessing the influence of robotic walking in this population is needed to confirm these preliminary results and to determine the optimal timing to introduce this intervention and the clinical implications of robotic walking for reducing fracture risk.

## Data Availability

The original contributions presented in the study are included in the article/supplementary material, further inquiries can be directed to the corresponding author/s.
